# No ‘cure’ within 12 years of diagnosis among breast cancer patients who are diagnosed via mammographic screening: women diagnosed in the West Midlands region of England 1989–2011

**DOI:** 10.1093/annonc/mdw408

**Published:** 2016-08-29

**Authors:** L. M. Woods, M. Morris, B. Rachet

**Affiliations:** Cancer Research UK Cancer Survival Group, Faculty of Epidemiology and Population Health, Department of Non-Communicable Disease Epidemiology, London School of Hygiene and Tropical Medicine, London, UK

**Keywords:** breast cancer, ‘cure’, deprivation, early diagnosis, screen-detection, population-based

## Abstract

**Background:**

We have previously reported that there is little evidence of population ‘cure’ among two populations of women diagnosed with invasive breast cancer. ‘Cure’ has not yet been examined in the context of screen-detection.

**Patients and methods:**

We examined cancer registry data on 19 800 women aged 50–70, diagnosed with a primary, invasive, non-metastatic breast cancer between 1 April 1989 and 31 March 2011 in the West Midlands region of England, linked to Hospital Episode Statistics (HES) and the National Breast Screening Service (NBSS). Follow-up was complete on all women up to 31 July 2012. Analyses were stratified by screening status, age, tumour stage, deprivation and ethnicity. We estimated net survival for the whole cohort and each subgroup. Population ‘cure’ was evaluated by fitting flexible parametric log-cumulative excess hazard regression models in which the excess hazard of breast cancer death was assumed to be equal to zero after a given follow-up time.

**Results:**

There was an overall lack of evidence for ‘cure’. Across all subgroups examined, the general pattern was that of a continuous decrease in net survival over time, with no obvious asymptotic tendency within 12 years of follow-up. Model-based analyses confirmed this observation.

**Conclusions:**

Despite dramatic improvements in survival over past decades, diagnosis with breast cancer remains associated with a small but persistent increased risk of death for all groups of women, including those whose cancer is detected asymptomatically. These findings are unlikely to be due to methodological inadequacies. Communication of these long-term consequences of breast cancer among women recently diagnosed and to those considering undergoing screening should take due consideration of these patterns.

## Abstract (precis)

Despite dramatic improvements in survival over past decades, diagnosis with breast cancer leads to a small but persistent, long-term increased risk of death for all groups of women. This is also true for those whose cancer is detected asymptomatically via screening mammography.

## introduction

**Figure 1.**
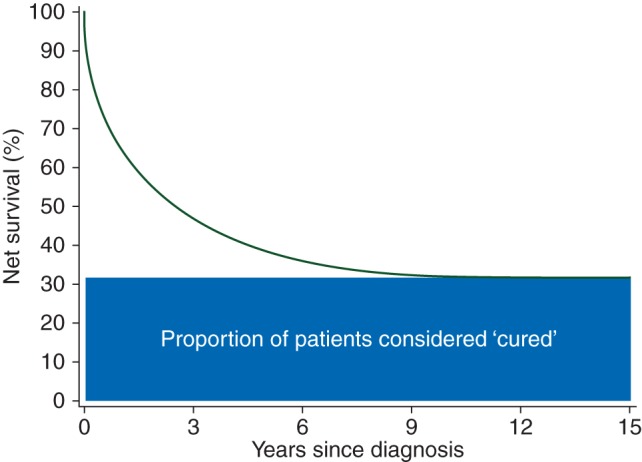
‘Cure’ in a hypothetical group of cancer patients.

Associated with the substantial and welcome increase in survival for the majority of cancer patients over the past 40 years [1–3] has been an increased interest in the statistical estimation of population ‘cure’ [4, 5]. ‘Cure’ in this context is the point at which a group of cancer patients is observed to have no excess mortality (due to their cancer) in comparison with the population from which they were drawn (Figure 1). At the point of ‘cure’, the group of cancer patients are no longer more likely to die than if they had never been diagnosed with cancer.

We previously found little evidence that this point of ‘cure’ was reached after 23 years of follow-up among two populations of women diagnosed with invasive breast cancer in England and Australia during the 1980–1995 [6]. Subsequent analyses have supported this conclusion [7, 8].

‘Cure’ among breast cancer patients has not yet been examined in the context of screen-detection. It is possible that micro-metastases, a likely candidate for the continued excess mortality seen in breast cancer patients overall [9], may be absent in the subpopulation diagnosed asymptomatically via screening. This subgroup would not then experience any long-term excess (cancer-related) mortality. This question is of great interest in the context of the recent review of the benefits and harms of mammographic screening [10] and the expansion of the screening age range in the UK to women aged 49–73 years [11].

Contrasting with survival, population ‘cure’ is independent of lead-time bias [12]. Indeed, the additional time afforded by early detection inflates cancer survival estimates at a given point in time after diagnosis, while it does not affect the proportion of patients who eventually display no long-term excess mortality.

We aim here to establish whether women who are diagnosed asymptomatically via screening display long-term excess mortality. We also analyse patterns by socioeconomic status and ethnicity to investigate whether these impact ‘cure’.

## materials and methods

### cohort selection

We examined women aged 50–70, diagnosed with a primary, invasive, non-metastatic breast cancer between 1 April 1989 and 31 March 2011 in the West Midlands region of England. Only those who had been continuously eligible for screening from the age of 50 onwards were included (described in detail elsewhere [13]). Cancer registry data on these individuals were obtained from the West Midlands Cancer Intelligence Unit and Breast Screening Quality Assurance Reference Centre [14]. Additional information was provided by Hospital Episode Statistics (HES) records individually linked to National Breast Screening Service (NBSS) data. Follow-up was complete on all women up to 31 July 2012.

### tumour stage

Information on tumour size, nodal involvement and presence of metastases was used to establish each woman's extent of disease at diagnosis, either localised (confined to the organ of origin) or regional (spread to adjacent muscle, organ, fat, connective tissue or regional lymph nodes). Those with distant metastases were excluded from all analyses *a priori*, since ‘cure’ was not a reasonable expectation for these women.

### deprivation

Deprivation was measured using the income domain of the English indices of deprivation for 2004, 2007 or 2010 [15–17]. These scores are derived from routine administrative data, pertaining to the years 2001, 2005 and 2008, respectively, for each of the 32 482 Lower Super Output Areas as defined at the 2001 census (LSOAs, ∼1500 people). The scores are categorised according to the quintiles of their national distribution. Each woman was assigned to one of five deprivation levels on the basis of her address of residence when diagnosed.

Our approach for deriving ethnicity information for this cohort has been described [13]. Briefly, data on each woman's ethnicity were gathered from self-reports given on admittance to hospital (from HES data, 83% of women), or where this was missing, on presentation for breast screening (from NBSS data, 7%). We imputed the remaining 10% of ethnicity data using name recognition software, Onomap [18]. This software matches the first and last names of the cohort patients with databases of names from different ethnicities.

### estimation of net survival and ‘cure’

We estimated net survival using the non-parametric Pohar Perme estimator [19] implemented in *stns:* software available for Stata 13. Net survival provides an estimate of survival from the cancer itself, adjusting for expected mortality from other causes, which was obtained from ethnic-specific life tables for England and Wales adjusted for deprivation [20].

We fitted flexible parametric log-cumulative excess hazard regression models [21] to estimate the age-adjusted excess hazard of breast cancer death. Models were fitted to follow-up times up to the 95th centile of (all) deaths. We assessed the linearity and time-dependence of age at diagnosis by the inclusion of restricted cubic splines, with the knots placed within the range of the data. Population ‘cure’ was then evaluated from the most parsimonious age-adjusted model by assuming that the excess hazard became equal to zero from a given time (as implemented in the software stpm2) [22, 23]. The final model was selected based on the lowest Akaike's information criterion (AIC) with a reduction of 3 or more in the AIC between successive models [24]. Where the difference between the ‘cure’ model and the age-adjusted model showed a reduction of 3 or more in the AIC, there was taken to be evidence of ‘cure’. The presence of ‘cure’ was also assessed by visual inspection of the survival curves.

### co-variables examined

Analyses were stratified by screening status (screen-detected/not-screen-detected). Additionally, we examined ‘cure’ by age (50–59/60–70 years), tumour stage (localised/regional), deprivation quintile [less deprived (quintiles 1 and 2)/more deprived (quintiles 3–5)] and ethnicity (White/Asian/Black). We also conducted a restricted analysis of localised cases only, by both age and deprivation.

## results

The analysis included 19 800 women who had a first primary malignant breast tumour which was not classified as distant at diagnosis (mean age 57.5 years, standard deviation = 5.0).

**Figure 2.**
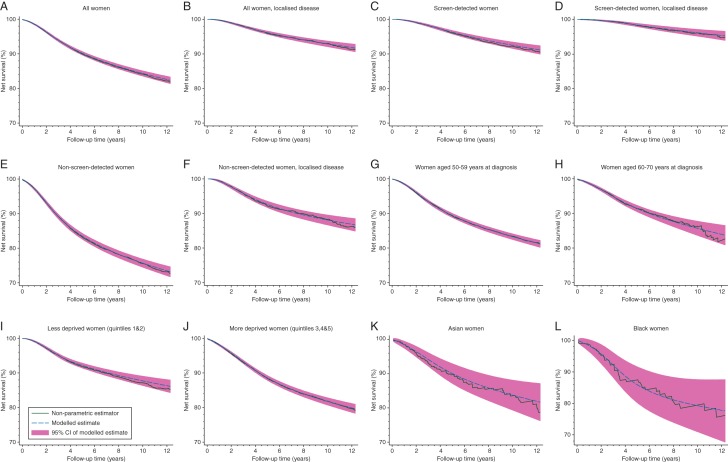
Non-parametric and modelled estimates of net survival up to 12 years following diagnosis. (A) All women. (B) All women, localised disease. (C) Screen-detected women. (D) Screen-detected women, localised disease. (E) Non-screen-detected women. (F) Non-screen-detected women, localised disease. (G) Women aged 50–59 years at diagnosis. (H) Women aged 60–70 years at diagnosis. (I) Less deprived women (quintiles 1 and 2). (J) More deprived women (quintiles 3–5). (K) Asian women. (L) Black women.

There was an overwhelming lack of evidence for ‘cure’. Despite high survival at 1, 5 and 10 years across the subgroups examined (defined by screening status, tumour stage, age, deprivation and ethnicity), there was a general pattern of a continuous decrease in net survival through time, with no obvious asymptotic tendency within 12 years (Figure 2, supplementary Figures S1–S3, available at *Annals of Oncology* online). The model-based analyses confirmed this observation; no ‘cure’ models were found to fit well for any subgroup examined (Table 1, supplementary Tables S1–S3, available at *Annals of Oncology* online).

**Table 1.** Evidence of ‘cure’ by screen-detection status: women diagnosed in the West Midlands region of England 1989–2011

	All			Screen-detected women			Non-screen-detected women		
	*n* (%)	Deaths (% of *n*)	Evidence of ‘cure’?^a^	*n* (%)	Deaths (% of *n*)	Evidence of ‘cure’?	*n* (%)	Deaths (% of *n*)	Evidence of ‘cure’?
All women	19 800 (100.0)	3153 (15.9)	No evidence	10 466 (100.0)	984 (9.4)	No evidence	9334 (100.0)	2169 (23.2)	No evidence
Age at diagnosis									
50–59 years	12 933 (65.3)	2316 (17.9)	No evidence	6563 (62.7)	699 (10.7)	No evidence	6370 (68.2)	1617 (25.4)	No evidence
60–69 years	6867 (34.7)	837 (12.2)	No evidence	3903 (37.3)	285 (7.3)	No evidence	2964 (31.8)	552 (18.6)	No evidence
Extent of disease at diagnosis^b^									
Localised	12 176 (61.5)	1121 (9.2)	No evidence	7548 (72.1)	499 (6.6)	No evidence	4628 (49.6)	622 (13.4)	No evidence
Regional	6364 (32.1)	1721 (27.0)	No convergence	2385 (22.8)	422 (17.7)	No evidence	3979 (42.6)	1299 (32.6)	No evidence
Ethnicity^c^									
White	19 040 (96.2)	3030 (15.9)	No evidence	10 087 (96.4)	949 (9.4)	No evidence	8953 (95.9)	2081 (23.2)	No evidence
Asian	572 (2.9)	85 (14.9)	No evidence	293 (2.8)	25 (8.5)	No convergence	279 (3.0)	60 (21.5)	No evidence
Black	188 (0.9)	38 (20.2)	No evidence	86 (0.8)	10 (11.6)	No convergence	102 (1.1)	28 (27.5)	No evidence
Deprivation quintile^d^									
Less deprived (1 and 2)	8592 (43.4)	1186 (13.8)	No evidence	4519 (43.2)	345 (7.6)	No evidence	4073 (43.6)	841 (20.6)	No evidence
More deprived (3–5)	11 190 (56.5)	1964 (17.6)	No evidence	5940 (56.8)	639 (10.8)	No evidence	5250 (56.2)	1325 (25.2)	No evidence
Among localised cases only	*n* = 12 176 (100.0)			*n* = 7548 (100.0)			*n* = 4628 (100.0)		
Age at diagnosis									
50–59 years	7701 (63.2)	796 (10.3)	No evidence	4576 (60.6)	335 (7.3)	No evidence	3125 (67.5)	461 (14.8)	No evidence
60–69 years	4475 (36.8)	325 (7.3)	No evidence	2972 (39.4)	164 (5.5)	No evidence	1503 (32.5)	161 (10.7)	No evidence
Deprivation quintile									
Less deprived (1 and 2)	5379 (44.2)	410 (7.6)	No evidence	3276 (43.4)	159 (4.9)	No evidence	2103 (45.4)	251 (11.9)	No evidence
More deprived (3–5)	6791 (55.8)	711 (10.5)	No evidence	4267 (56.5)	340 (8.0)	No evidence	2524 (54.5)	371 (14.7)	No evidence

^a^As determined by the difference in the AIC: reduction of 3 or more = evidence of ‘cure’; increase or a reduction of <3 = no evidence of ‘cure’; ‘cure’ model unable to converge = ‘no convergence’.

^b^Unstaged cancers (*n* = 1260) were excluded from extent-specific analyses.

^c^Individual ethnicity: White includes all categories other than Asian and Black (see text).

^d^Quintile of the IMD income domain score of the woman's LSOA of residence at diagnosis (see text). Women with missing data were excluded (*n* = 18).

Models did not always converge. Among the screen-detected group, parametric survival models could not be fitted for either Black or Asian women due to small numbers of patients and deaths in these groups. Fitting an asymptote to the age-adjusted model for women with regional disease also proved unachievable. For these women, ‘cure’ was not assessed using the modelling approach.

**Figure 3.**
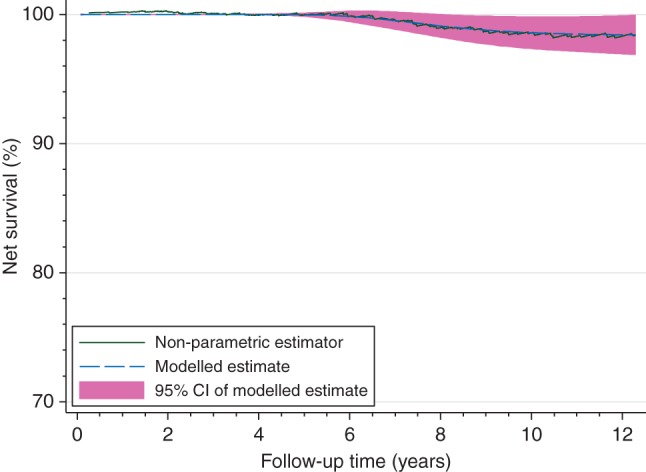
Non-parametric and modelled estimates of net survival up to 12 years following diagnosis: less deprived women with localised disease whose tumour was screen-detected.

The one subgroup which displayed a different pattern was affluent women screen-detected with localised disease (Figure 3). Here, survival was very high, in excess of 98% after 10 years. The net survival curve tended slightly towards an asymptote, and the model also confirmed a flattening of the curve. The ‘cure’ model did not, however, display a better fit than the age-adjusted model alone.

## discussion

We have shown that there is a persistent lack of ‘cure’ among this cohort of middle-aged women diagnosed with breast cancer for all sociodemographic groups, even if their cancer is localised and/or detected via screening. Elevated mortality for all groups persists beyond the 10th anniversary of diagnosis.

There was suggestive, but weak, evidence of ‘cure’ around 12 years after diagnosis for less deprived women with localised disease whose cancer was detected via screening. Although the net survival curve tended to level from the 11th year following diagnosis, the model-based analysis did not support the hypothesis that ‘cure’ was present, however.

### strengths and limitations

Our approach has several strengths in comparison with previous studies. Life tables specific, not just to the deprivation profile of this population, but also its ethnic mix, were applied to obtain the most accurate estimates of expected mortality in this setting. Screening status was established on the basis of individually linked data, and we restricted the cohort to women whom we know to have been invited for screening from their 50th birthday onwards. The influence of screen-detection upon ‘cure’ is not thus obscured by older women attending screening for the first time at ages over 50 years. We used flexible models to test the existence of ‘cure’, rather than one which assumes its existence, as necessitated by other methods [5, 7]. This means that the presence of the ‘cured’ proportion can therefore be formally evaluated. As the AIC assesses the whole curve, however, while for ‘cure’ the tail of the curve (where there are more sparse data) is most important, caution must be exercised in relying solely on this evaluation. To this end, the need for visual inspection of the net survival curves continues to be emphasised [7], which we did, with the same conclusions.

There are limitations of our analysis. Breast cancer survival is high, thus there were a relatively small number of deaths in our data. We therefore restricted all analyses to the first 95% of deaths to reduce poor model fit in particular at the end of follow-up.

A related concern is the inappropriateness of the AIC for evaluating ‘cure’ models [7], because the AIC is less sensitive to the portion of follow-up where ‘cure’ occurs. However, deaths here occurred at a steadily decreasing rate throughout follow-up, with a not-so-skewed distribution of times to death (mean time to death 4.34 years, median 3.24 years, inter-quartile range 1.55–6.06).

We have previously evaluated cure up to 23 years after diagnosis. Although the maximum follow-up of the present cohort was similar, our cautious restriction of examining ‘cure’ only up to the 95th centile deaths meant that the effective follow-up was much shorter: 12.3 years. It is possible that ‘cure’ could be reached by survivors remaining after this time, although the trajectory of the survival curves suggests that this is very unlikely.

We excluded confirmed distant (metastatic) cancers from our analyses since we did not reasonably expect ‘cure’ to be attained for these patients. However, because we also included unstaged tumours in the overall analyses, survival reported here for all patients is a slight underestimate of the survival of women with localised or regional tumours.

### possible causal explanations

Persistent excess mortality due to cancer among screen-detected women into the second decade following their diagnosis seems unlikely to be due to treatment inadequacies at the time of the initial diagnosis, but rather more likely to be due to long-term effects of either the cancer itself or of its treatment, and, or the distinctive natural history of this malignancy. For example, some women whose disease is apparently localised at diagnosis harbour micro-metastatic disease: it is possible that this is also the case among women who are asymptomatic and screen-detected.

The data available did not allow us to investigate ‘cure’ by molecular subtype of breast cancer (e.g. luminal A or B, triple negative, HER2). Certain subtypes may have already metastasised even when the tumour itself is localised [25, 26], which could partly explain the lack of cure in the cohort overall.

A further hypothesis has been proposed that the act of breast cancer surgery itself provokes the activation of latent micro-metastases [27, 28]. However, this mechanism has been suggested only among pre-menopausal women, whereas women under 50 were not included in this study.

### public health considerations

The public health implications of these findings are twofold. First, our analysis strongly suggests that despite very high survival overall, women diagnosed with breast cancer experience a continuing risk of death from cancer beyond the 10th anniversary of their diagnosis, and that this occurs irrespective of their extent of disease at diagnosis. This has implications for the way in which clinicians, policy makers and public health professionals communicate with patients regarding the long-term prognosis to women newly diagnosed with breast cancer. In particular, data such as these question whether a woman diagnosed once with breast cancer can be considered to be disease-free, and increases the importance of using the correct language when communicating with those who have previously been treated for breast cancer [29, 30]. Second, since the pattern is consistent for both screen-detected and non-screen-detected women, our data suggest that screening does not afford protection from long-term excess mortality, even though it is associated with an important and significant survival advantage at all times since diagnosis, independent of lead-time bias [13]. Communication of this important and unique feature of breast cancer to those women considering screening and diagnosed via screening should also be carefully considered.

### conclusion

Our analyses have shown an overwhelming lack of evidence for ‘cure’ in our cohort of breast cancer patients. We have demonstrated continued excess mortality up to 12 years after diagnosis, irrespective of age, screening status, stage of disease, ethnicity or deprivation status. These findings are unlikely to be due to methodological inadequacies. Despite high and continually increasing survival among middle-aged women diagnosed in the UK, breast cancer leads to a tiny, but persistent, increased risk of death for all groups of women, including those whose cancer is detected asymptomatically. Communication of the long-term consequences of breast cancer among women recently diagnosed and to those considering undergoing screening should take due consideration of these patterns.

## funding

This work was supported by the National Awareness and Early Diagnosis Initiative (NAEDI) (C23409/A14031 to MM) and by Cancer Research UK (C23409/A11415 to LMW and C1336/A11700 to BR).

## disclosure

The authors have declared no conflicts of interest.

## Supplementary Material

Supplementary Data
