# Supplementary material for: No ‘cure’ within 12 years of diagnosis among breast cancer patients who are diagnosed via mammographic screening: women diagnosed in the West Midlands region of England 1989–2011
Source: Ann Oncol. 2016 Aug 29;27(11):2025–31. doi: 10.1093/annonc/mdw408 (PMC5091325; doi:10.1093/annonc/mdw408)
Supplement: Supplementary Data [file supp_27_11_2025__index.html]

No ‘cure’ within 12 years of diagnosis among breast cancer patients who are diagnosed via mammographic screening: women diagnosed in the West Midlands region of England 1989–2011 — No ‘cure’ within 12 years of diagnosis among breast cancer patients who are diagnosed via mammographic screening: women diagnosed in the West Midlands region of England 1989–2011 — Supplementary Data 

# No ‘cure’ within 12 years of diagnosis among breast cancer patients who are diagnosed via mammographic screening: women diagnosed in the West Midlands region of England 1989–2011

## Supplementary Data

Supplementary Data

- Supplementary Data - Docx file
- Supplementary Figure 1 - jpg file
- Supplementary Figure 2 - jpg file
- Supplementary Figure 3 - jpg file
- Supplementary Table 1 - docx file
- Supplementary Table 2 - docx file
- Supplementary Table 3 - docx file
