# Supplementary material for: No ‘cure’ within 12 years of diagnosis among breast cancer patients who are diagnosed via mammographic screening: women diagnosed in the West Midlands region of England 1989–2011
Source: Ann Oncol. 2016 Aug 29;27(11):2025–31. doi: 10.1093/annonc/mdw408 (PMC5091325; doi:10.1093/annonc/mdw408)
Supplement: Supplementary Data [file supp_mdw408_mdw408supp.docx]

**Table title**

**Table titles for supplementary material (published online only, providing full details of all models fitted)**

**Supplementary Material Table S1: Evidence of ‘cure’ including modelling details: women diagnosed in the West Midlands region of England 1989-2011**

**Supplementary Material Table S2: Evidence of ‘cure’ including modelling details: women diagnosed via screening mammography in the West Midlands region of England 1989-2011**

**Supplementary Material Table S3: Evidence of ‘cure’ including modelling details: women not diagnosed via screening mammography in the West Midlands region of England 1989-2011**

**Figure legends for supplementary material (published online only, providing a full complement of all results)**

**Supplementary Material Figure S1: Non-parametric and modelled estimates of net survival: All women**

a) All

b) 50-59 years

c) 60-70 years

d) Localised disease

e) Regional disease

f) Asian

g) Black

h) White

i) Less deprived

j) More deprived

k) Localised disease, 50-59 years

l) Localised disease, 60-70 years

m) Localised disease, less deprived

n) Localised disease, more deprived

**Supplementary Material Figure S2: Non-parametric and modelled estimates of net survival: Screen-detected women**

a) All

b) 50-59 years

c) 60-70 years

d) Localised disease

e) Regional disease

f) Less deprived

g) More deprived

h) Localised disease, 50-59 years

i) Localised disease, 60-70 years

j) Localised disease, less deprived

k) Localised disease, more deprived

**Supplementary Material Figure S3: Non-parametric and modelled estimates of net survival: Non-screen-detected women**

a) All

b) 50-59 years

c) 60-70 years

d) Localised disease

e) Regional disease

f) White

g) Black

h) Asian

i) Less deprived

j) More deprived

k) Localised disease, 50-59 years

l) Localised disease, 60-70 years

m) Localised disease, less deprived

n) Localised disease, more deprived
