# Supplementary figures and images for: No ‘cure’ within 12 years of diagnosis among breast cancer patients who are diagnosed via mammographic screening: women diagnosed in the West Midlands region of England 1989–2011
Source: Ann Oncol. 2016 Aug 29;27(11):2025–31. doi: 10.1093/annonc/mdw408 (PMC5091325; doi:10.1093/annonc/mdw408)

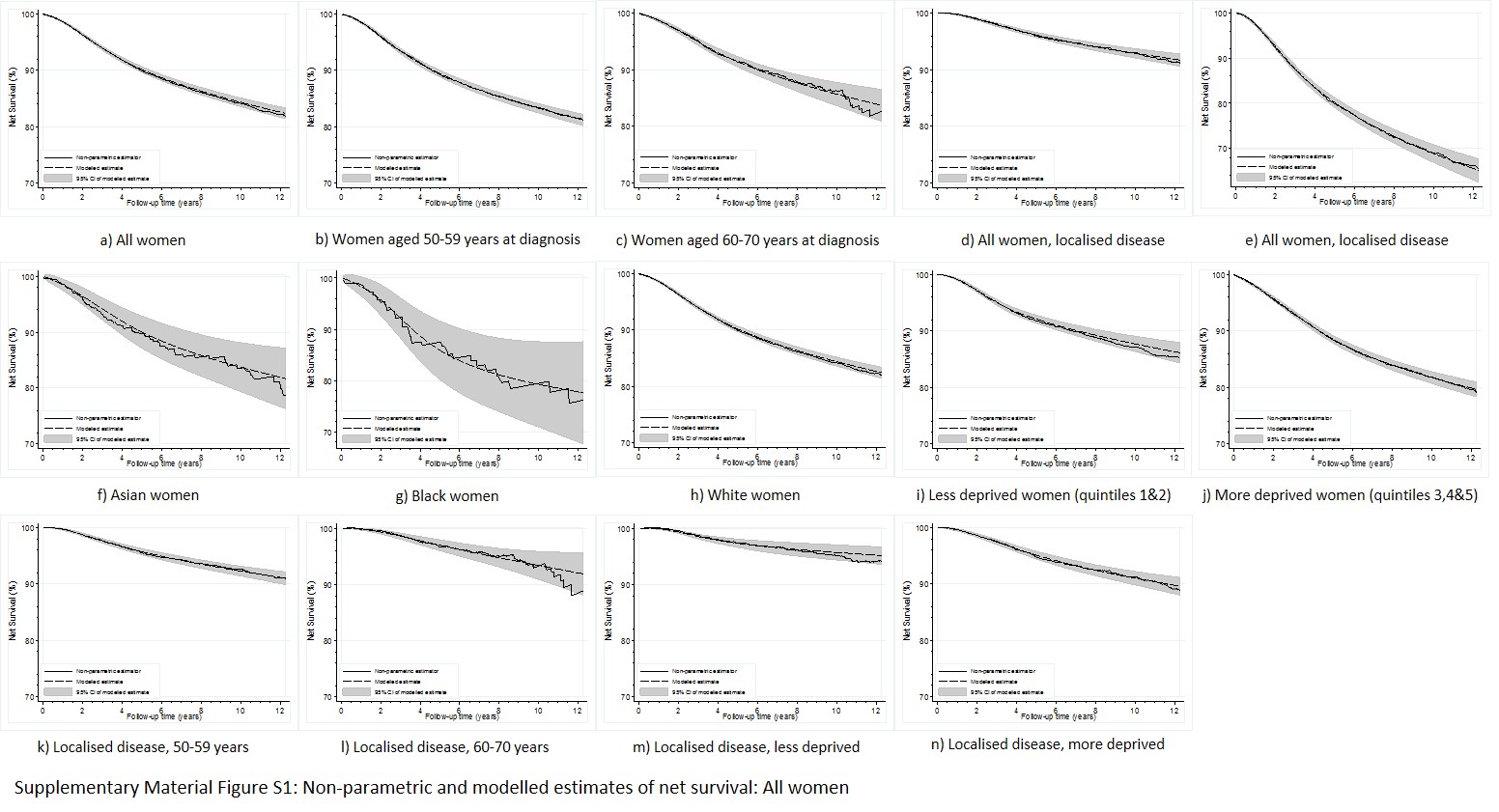

Supplement: Supplementary Data [file supp_mdw408_mdw408supp_fig1.jpg]

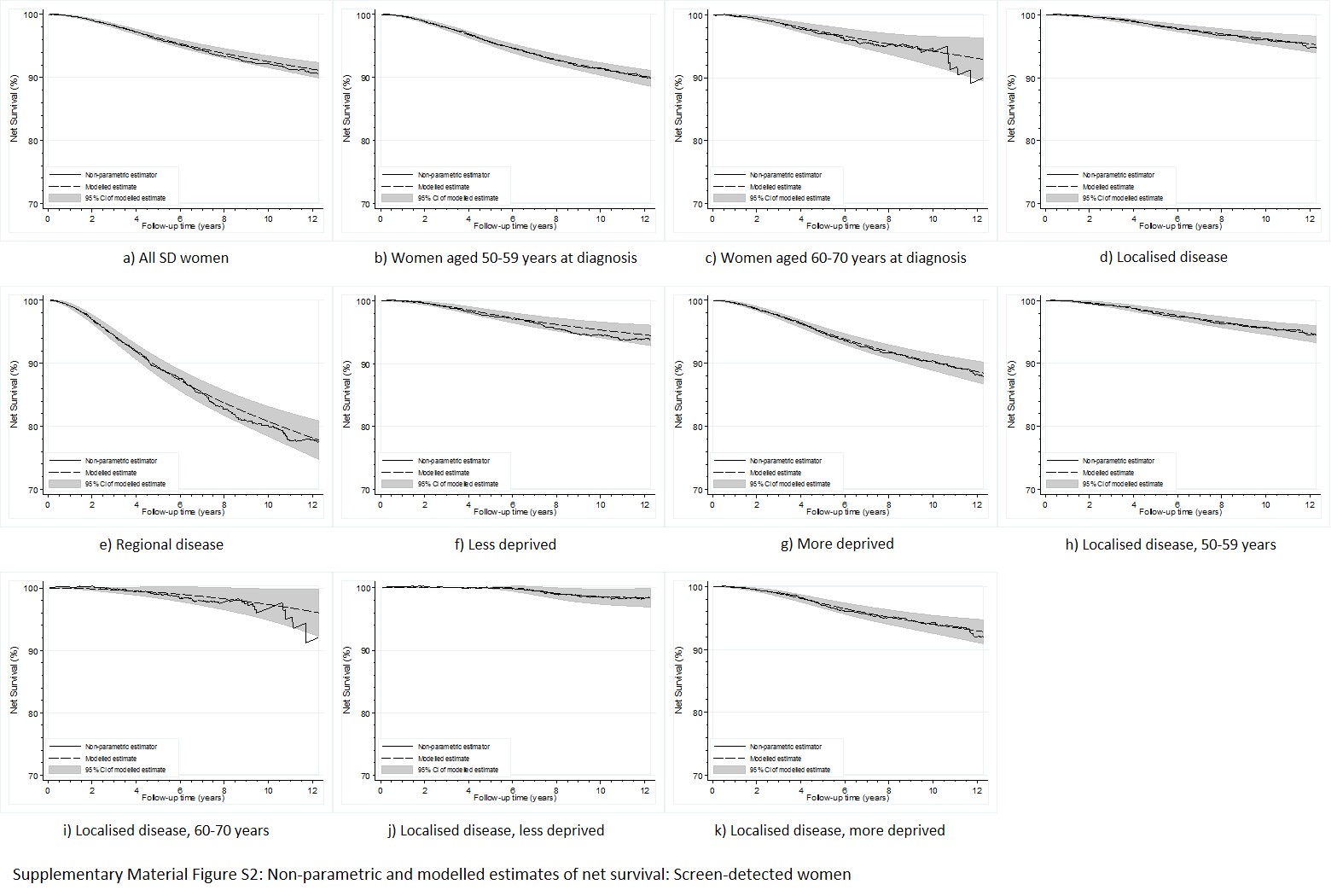

Supplement: Supplementary Data [file supp_mdw408_mdw408supp_fig2.jpg]

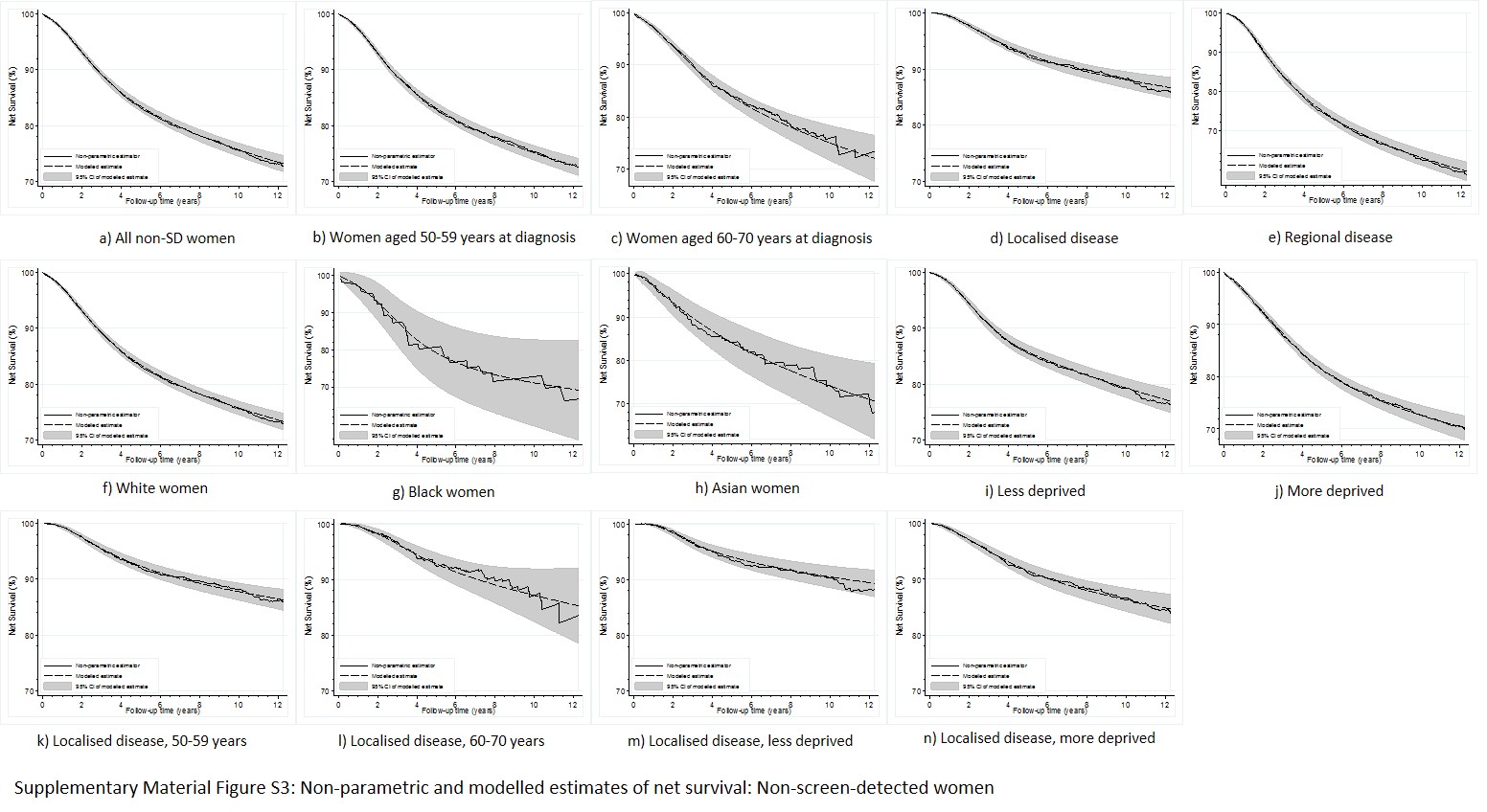

Supplement: Supplementary Data [file supp_mdw408_mdw408supp_fig3.jpg]
