# Supplementary material for: No ‘cure’ within 12 years of diagnosis among breast cancer patients who are diagnosed via mammographic screening: women diagnosed in the West Midlands region of England 1989–2011
Source: Ann Oncol. 2016 Aug 29;27(11):2025–31. doi: 10.1093/annonc/mdw408 (PMC5091325; doi:10.1093/annonc/mdw408)
Supplement: Supplementary Data [file supp_mdw408_mdw408supp_table1.docx]

**Supplementary Material Table S1: Evidence of ‘cure’ including modelling details: women diagnosed in the West Midlands region of England 1989-2011**

|  |  |  | **All** | | | | |
| --- | --- | --- | --- | --- | --- | --- | --- |
|  |  |  | **N (%)** | **Deaths (% of N)** | **Model description^1^** | **Difference in AIC^2^** | **Evidence of 'cure'?^3^** |
|  |  |  |  |  |  |  |  |
|  |  |  |  |  |  |  |  |
| **All women** | | | **19,800 (100.0)** | **3,153 (15.9)** | **Non-linear with 3df; Not time-dependent** | **-89.85** | **No evidence** |
|  |  |  |  |  |  |  |  |
|  |  |  |  |  |  |  |  |
|  | Age at diagnosis | |  |  |  |  |  |
|  |  | *50-59 years* | 12,933 (65.3) | 2,316 (17.9) | Non-linear with 2df; Not time-dependent | -75.27 | No evidence |
|  |  | *60-69 years* | 6,867 (34.7) | 837 (12.2) | Non-linear with 2df; Not time-dependent | -12.86 | No evidence |
|  | Extent of disease at diagnosis^4^ | |  |  |  |  |  |
|  |  | *Localised* | 12,176 (61.5) | 1,121 (9.2) | Linear; Not time-dependent | -30.1 | No evidence |
|  |  | *Regional* | 6,364 (32.1) | 1,721 (27.0) | - | - | No convergence |
|  | Ethnicity^5^ | |  |  |  |  |  |
|  |  | *White* | 19,040 (96.2) | 3,030 (15.9) | Non-linear with 3df; Not time-dependent | -84.62 | No evidence |
|  |  | *Asian* | 572 (2.9) | 85 (14.9) | Non-linear with 2df; Not time-dependent | -4.73 | No evidence |
|  |  | *Black* | 188 (0.9) | 38 (20.2) | Linear; Not time-dependent | 1.24 | No evidence |
|  | Deprivation quintile^6^ | |  |  |  |  |  |
|  |  | *Less deprived (1&2)* | 8,592 (43.4) | 1,186 (13.8) | Non-linear with 3df; Time-dependent with 2df | -45.21 | No evidence |
|  |  | *More deprived (3,4&5)* | 11,190 (56.5) | 1,964 (17.6) | Linear; Not time-dependent | -78.03 | No evidence |
|  |  |  |  |  |  |  |  |
|  |  |  |  |  |  |  |  |
| ***Amongst localised cases only*** | | | ***N=12,176 (100.0)*** | |  |  |  |
|  | Age at diagnosis | |  |  |  |  |  |
|  |  | *50-59 years* | 7,701 (63.2) | 796 (10.3) | Linear; Not time-dependent | -24.94 | No evidence |
|  |  | *60-69 years* | 4,475 (36.8) | 325 (7.3) | Linear; Not time-dependent | -3.31 | No evidence |
|  | Deprivation quintile^6^ | |  |  |  |  |  |
|  |  | *Less deprived (1&2)* | 5,379 (44.2) | 410 (7.6) | Non-linear with 3df; Time-dependent with 2df | -12.62 | No evidence |
|  |  | *More deprived (3,4&5)* | 6,791 (55.8) | 711 (10.5) | Linear; Not time-dependent | -22.58 | No evidence |
|  |  |  |  |  |  |  |  |
|  |  |  |  |  |  |  |  |
| **Footnotes to Supplementary Material tables S1, S2 and S3** | | | | |  |  |  |
| ^1^Age effects included in the age-adjusted model, with degrees of freedom for each effect | | | | | |  |  |
| ^2^Difference in AIC between 'cure' and age-adjusted model where 'cure' was not assumed | | | | | |  |  |
| ^3^As determined by the difference in the AIC: reduction of 3 or more = "Evidence of 'cure'"; increase or a reduction of less than 3 = "No evidence of 'cure'"; 'cure' model unable to converge = "No convergence". | | | | | | | |
| ^4^Unstaged cancers (N=1,260) were excluded from extent-specific analyses. | | | | |  |  |  |
| ^5^Individual ethnicity: White includes all categories other than Asian and Black (see text). | | | | | |  |  |
| ^6^Quintile of the IMD income domain score of the woman's LSOA of residence at diagnosis (see text). Women with missing data were excluded (N=18). | | | | | | |  |
