# Supplementary material for: No ‘cure’ within 12 years of diagnosis among breast cancer patients who are diagnosed via mammographic screening: women diagnosed in the West Midlands region of England 1989–2011
Source: Ann Oncol. 2016 Aug 29;27(11):2025–31. doi: 10.1093/annonc/mdw408 (PMC5091325; doi:10.1093/annonc/mdw408)
Supplement: Supplementary Data [file supp_mdw408_mdw408supp_table2.docx]

**Supplementary Material Table S2: Evidence of ‘cure’ including modelling details: women diagnosed via screening mammography in the West Midlands region of England 1989-2011**

|  |  |  | **Screen-detected women** | | | | | | | |
| --- | --- | --- | --- | --- | --- | --- | --- | --- | --- | --- |
|  |  |  | **N (%)** | **Deaths (% of N)** | **Model description^1^** | | **Difference in AIC^2^** | | **Evidence of 'cure'?^3^** | |
|  |  |  |  |  |  | |  | |  | |
|  |  |  |  |  |  | |  | |  | |
| **All women** | | | **10,466 (100.0)** | **984 (9.4)** | **Non-linear with 2df; Not time-dependent** | | **-19.55** | | **No evidence** | |
|  |  |  |  |  |  | |  | |  | |
|  |  |  |  |  |  | |  | |  | |
|  | Age at diagnosis | |  |  |  | |  | |  | |
|  |  | *50-59 years* | 6,563 (62.7) | 699 (10.7) | Linear; Not time-dependent | | -14.93 | | No evidence | |
|  |  | *60-69 years* | 3,903 (37.3) | 285 (7.3) | Linear; Not time-dependent | | -1.35 | | No evidence | |
|  | Extent of disease at diagnosis^4^ | |  |  |  | |  | |  | |
|  |  | *Localised* | 7,548 (72.1) | 499 (6.6) | Linear; Not time-dependent | | -6.94 | | No evidence | |
|  |  | *Regional* | 2,385 (22.8) | 422 (17.7) | Non-linear with 3df; Not time-dependent | | -10.35 | | No evidence | |
|  | Ethnicity^5^ | |  |  |  | |  | |  | |
|  |  | *White* | 10,087 (96.4) | 949 (9.4) | Linear; Not time-dependent | | -19.76 | | No evidence | |
|  |  | *Asian* | 293 (2.8) | 25 (8.5) | - | | - | | No convergence | |
|  |  | *Black* | 86 (0.8) | 10 (11.6) | - | | - | | No convergence | |
|  | Deprivation quintile^6^ | |  |  |  | |  | |  | |
|  |  | *Less deprived (1&2)* | 4,519 (43.2) | 345 (7.6) | Non-linear with 2df; Not time-dependent | | 1.04 | | No evidence | |
|  |  | *More deprived (3,4&5)* | 5,940 (56.8) | 639 (10.8) | Linear; Not time-dependent | | -19.01 | | No evidence | |
|  |  |  |  |  |  | |  | |  | |
|  |  |  |  |  |  | |  | |  | |
| ***Amongst localised cases only*** | | | ***N=7,548 (100.0)*** | |  | |  | |  | |
|  | Age at diagnosis | |  |  |  | |  | |  | |
|  |  | *50-59 years* | 4,576 (60.6) | 335 (7.3) | Linear; Not time-dependent | | -5.33 | | No evidence | |
|  |  | *60-69 years* | 2,972 (39.4) | 164 (5.5) | Linear; Not time-dependent | | -1.56 | | No evidence | |
|  | Deprivation quintile^6^ | |  |  |  | |  | |  | |
|  |  | *Less deprived (1&2)* | 3,276 (43.4) | 159 (4.9) | Linear; Not time-dependent | | 1.97 | | No evidence | |
|  |  | *More deprived (3,4&5)* | 4,267 (56.5) | 340 (8.0) | Linear; Not time-dependent | | -9.33 | | No evidence | |
|  |  |  |  |  |  | |  | |  | |
|  |  |  |  |  |  | |  | |  | |
| **Footnotes to Supplementary Material tables S1, S2, and S3** | | | | | |  | |  | |  |
| ^1^Age effects included in the age-adjusted model, with degrees of freedom for each effect | | | | | | | |  | |  |
| ^2^Difference in AIC between 'cure' and age-adjusted model where 'cure' was not assumed | | | | | | | |  | |  |
| ^3^As determined by the difference in the AIC: reduction of 3 or more = "Evidence of 'cure'"; increase or a reduction of less than 3 = "No evidence of 'cure'"; 'cure' model unable to converge = "No convergence". | | | | | | | | | | |
| ^4^Unstaged cancers (N=1,260) were excluded from extent-specific analyses. | | | | | |  | |  | |  |
| ^5^Individual ethnicity: White includes all categories other than Asian and Black (see text). | | | | | | | |  | |  |
| ^6^Quintile of the IMD income domain score of the woman's LSOA of residence at diagnosis (see text). Women with missing data were excluded (N=18). | | | | | | | | | |  |
