# Supplementary material for: No ‘cure’ within 12 years of diagnosis among breast cancer patients who are diagnosed via mammographic screening: women diagnosed in the West Midlands region of England 1989–2011
Source: Ann Oncol. 2016 Aug 29;27(11):2025–31. doi: 10.1093/annonc/mdw408 (PMC5091325; doi:10.1093/annonc/mdw408)
Supplement: Supplementary Data [file supp_mdw408_mdw408supp_table3.docx]

**Supplementary Material Table S3: Evidence of ‘cure’ including modelling details: women not diagnosed via screening mammography in the West Midlands region of England 1989-2011**

|  |  |  | **Non-screen-detected women** | | | | | | | |
| --- | --- | --- | --- | --- | --- | --- | --- | --- | --- | --- |
|  |  |  | **N (%)** | **Deaths (% of N)** | **Model description^1^** | | **Difference in AIC^2^** | | **Evidence of 'cure'?^3^** | |
|  |  |  |  |  |  | |  | |  | |
|  |  |  |  |  |  | |  | |  | |
| **All women** | | | **9,334 (100.0)** | **2,169 (23.2)** | **Linear; Not time-dependent** | | **-86.93** | | **No evidence** | |
|  |  |  |  |  |  | |  | |  | |
|  |  |  |  |  |  | |  | |  | |
|  | Age at diagnosis | |  |  |  | |  | |  | |
|  |  | *50-59 years* | 6,370 (68.2) | 1,617 (25.4) | Non-linear with 3df; Time-dependent with 2df | | -80.52 | | No evidence | |
|  |  | *60-69 years* | 2,964 (31.8) | 552 (18.6) | Non-linear with 2df; Not time-dependent | | -11.36 | | No evidence | |
|  | Extent of disease at diagnosis^4^ | |  |  |  | |  | |  | |
|  |  | *Localised* | 4,628 (49.6) | 622 (13.4) | Linear; Not time-dependent | | -21.36 | | No evidence | |
|  |  | *Regional* | 3,979 (42.6) | 1,299 (32.6) | Linear; Not time-dependent | | -72.11 | | No evidence | |
|  | Ethnicity^5^ | |  |  |  | |  | |  | |
|  |  | *White* | 8,953 (95.9) | 2,081 (23.2) | Linear; Not time-dependent | | -79.44 | | No evidence | |
|  |  | *Asian* | 279 (3.0) | 60 (21.5) | Linear; Not time-dependent | | -7.39 | | No evidence | |
|  |  | *Black* | 102 (1.1) | 28 (27.5) | Linear; Not time-dependent | | -0.58 | | No evidence | |
|  | Deprivation quintile^6^ | |  |  |  | |  | |  | |
|  |  | *Less deprived (1&2)* | 4,073 (43.6) | 841 (20.6) | Linear; Not time-dependent | | -33.64 | | No evidence | |
|  |  | *More deprived (3,4&5)* | 5,250 (56.2) | 1,325 (25.2) | Non-linear with 2df; Time-dependent with 2df | | -68.55 | | No evidence | |
|  |  |  |  |  |  | |  | |  | |
|  |  |  |  |  |  | |  | |  | |
| ***Amongst localised cases only*** | | | ***N=4,628 (100.0)*** | |  | |  | |  | |
|  | Age at diagnosis | |  |  |  | |  | |  | |
|  |  | *50-59 years* | 3,125 (67.5) | 461 (14.8) | Linear; Not time-dependent | | -18.45 | | No evidence | |
|  |  | *60-69 years* | 1,503 (32.5) | 161 (10.7) | Linear; Not time-dependent | | -1.77 | | No evidence | |
|  | Deprivation quintile^6^ | |  |  |  | |  | |  | |
|  |  | *Less deprived (1&2)* | 2,103 (45.4) | 251 (11.9) | Non-linear with 2df; Not time-dependent | | -9.51 | | No evidence | |
|  |  | *More deprived (3,4&5)* | 2,524 (54.5) | 371 (14.7) | Linear; Not time-dependent | | -12.83 | | No evidence | |
|  |  |  |  |  |  | |  | |  | |
|  |  |  |  |  |  | |  | |  | |
| **Footnotes to Supplementary Material tables S1, S2, and S3** | | | | | |  | |  | |  |
| ^1^Age effects included in the age-adjusted model, with degrees of freedom for each effect | | | | | | | |  | |  |
| ^2^Difference in AIC between 'cure' and age-adjusted model where 'cure' was not assumed | | | | | | | |  | |  |
| ^3^As determined by the difference in the AIC: reduction of 3 or more = "Evidence of 'cure'"; increase or a reduction of less than 3 = "No evidence of 'cure'"; 'cure' model unable to converge = "No convergence". | | | | | | | | | | |
| ^4^Unstaged cancers (N=1,260) were excluded from extent-specific analyses. | | | | | |  | |  | |  |
| ^5^Individual ethnicity: White includes all categories other than Asian and Black (see text). | | | | | | | |  | |  |
| ^6^Quintile of the IMD income domain score of the woman's LSOA of residence at diagnosis (see text). Women with missing data were excluded (N=18). | | | | | | | | | |  |
